# Genetic and environmental melanoma models in fish

**DOI:** 10.1111/j.1755-148X.2010.00693.x

**Published:** 2010-03-08

**Authors:** E Elizabeth Patton, David L Mitchell, Rodney S Nairn

**Affiliations:** 1 Institute for Genetics and Molecular Medicine, MRC Human Genetics Unit and The Division of Cancer Research, The University of EdinburghEdinburgh, UK; 2 Department of Carcinogenesis, Science Park-Research Division, The University of Texas M.D. Anderson Cancer CenterSmithville, TX, USA; 3 Graduate School of Biomedical Sciences, The University of TexasHouston, TX, USA

**Keywords:** melanoma, *Xiphophorus*, zebrafish, medaka, UV

## Abstract

Experimental animal models are extremely valuable for the study of human diseases, especially those with underlying genetic components. The exploitation of various animal models, from fruitflies to mice, has led to major advances in our understanding of the etiologies of many diseases, including cancer. Cutaneous malignant melanoma is a form of cancer for which both environmental insult (i.e., UV) and hereditary predisposition are major causative factors. Fish melanoma models have been used in studies of both spontaneous and induced melanoma formation. Genetic hybrids between platyfish and swordtails, different species of the genus *Xiphophorus*, have been studied since the 1920s to identify genetic determinants of pigmentation and melanoma formation. Recently, transgenesis has been used to develop zebrafish and medaka models for melanoma research. This review will provide a historical perspective on the use of fish models in melanoma research, and an updated summary of current and prospective studies using these unique experimental systems.

## Gene-environment interactions and animal melanoma models

Carcinogenesis is a complex, multistage process driven by genetic and environmental factors. Melanoma is a salient example of the complexity of gene-environment interactions in carcinogenesis. Cutaneous malignant melanoma (CMM) is a deadly form of cancer which shows an alarming increase in incidence in the US and worldwide (Linos et al., 2009). Dissecting apart the genetic from the environmental elements of its complex etiology is important to understanding its causes and reversing this trend. Although it is recognized that sunlight is the major environmental cause of CMM, it is also clear that heredity is a very strong predisposing factor (Bishop et al., 2007; Chin et al., 2006; Rivers, 2004). For example, there are hereditary conditions such as familial atypical multiple mole-melanoma (FAMMM) syndrome (Bergman et al., 1992) as well as epidemiological data indicating that melanoma is one of the most familial cancers (Begg et al., 2004; Chaudru et al., 2004; Hemminki et al., 2003; Kerber and O’Brien, 2005; Rutter et al., 2004). Such studies of melanoma formation in human populations are necessarily retrospective, and therefore animal melanoma models are invaluable tools in which genetic and environmental components can be recognized and experimentally isolated.

Mammalian models are of obvious utility in cancer research, since they have the advantage of being physiologically most similar to humans, with directly comparable cell lineage and differentiation pathways. However, in many instances non-mammalian models, particularly genetic models, have convincingly demonstrated their value in cancer research (Friend, 1993). Fish models have been used extensively to study a variety of tumors, including hematological and liver cancers, various sarcomas, melanoma and other malignancies (Amatruda and Patton, 2008; Amatruda et al., 2002; Bailey et al., 1996; Bunton, 1996; Walter and Kazianis, 2001). Some advantages of fish models in cancer research include their high fecundity and often short breeding cycles to produce large numbers of progeny, cost efficiency, and easy exposure to carcinogens (Amatruda and Patton, 2008; Stern and Zon, 2003). More importantly, teleosts span the evolutionary distance between mammals and lower eukaryotic model organisms such as *Drosophila* and *C. elegans.* In terms of genomics, fish offer an enormously diverse range of genome size and complexity (Muller, 2005), and genetic tools including extensive and detailed gene maps and genome sequences have been developed (Froschauer et al., 2002; Hubbard et al., 2009; Kazianis et al., 2004b; Knapik et al., 1998; Schartl et al., 2004; Walter et al., 2004, 2006). Developmental and other types of mutants are available in well-characterized fish lines (Amsterdam and Hopkins, 2006; Patton and Zon, 2001). Innovative experimental tools such as morpholinos for gene knock-down (Nasevicius and Ekker, 2000), sophisticated transgenesis (Amsterdam and Hopkins, 2006; Esengil and Chen, 2008; Kwan et al., 2007), high throughput screening of mutants (Amsterdam and Hopkins, 2006; Patton and Zon, 2001) and for the pharmacological effects of small molecules (Zon and Peterson, 2005) are available. For all these reasons, fish models are increasingly being used in cancer research, not only as an adjunct to mammalian models, but in many cases because they accommodate the most robust experimental approaches to a particular scientific problem.

For melanoma research, a variety of non-rodent models has been studied including *Drosophila* (Hanratty and Ryerse, 1981), swine (Greene et al., 1997; Millikan et al., 1974), horse (Fleury et al., 2000; Rosengren Pielberg et al., 2008), and the marsupial *Monodelphis domestica* (Ley, 2002). Rodent melanoma models include the Syrian hamster (Fortner and Allen, 1958), hairless mouse (Kligman and Elenitsas, 2001) and several transgenic mouse lines (reviewed in Chin, 2003; Noonan et al., 2003), which have been used extensively to study UV-induced melanoma formation. Unlike in humans (and fish), murine melanocytes are confined to the hair follicles and not distributed throughout the epidermis, and mice are very refractory to melanoma induction by UV except in some transgenic models (Noonan et al., 2003). The most venerable experimental melanoma model, *Xiphophorus*, has been studied for over eight decades and actually constitutes a collection of genetic models useful for investigating both spontaneous and induced melanoma formation (Nairn et al., 2001; Walter and Kazianis, 2001). Recently, transgenic fish melanoma models have been developed in the zebrafish *Danio rerio* (Patton et al., 2005) and in medaka, *Oryzias latipes* (Schartl et al., 2010). This review will describe the contributions of fish melanoma models to our current understanding of melanoma formation, and prospects for future research using these unique experimental organisms.

## *Xiphophorus* melanoma models

### Early genetic studies of *Xiphophorus* melanomas

In the late 1920s, it was observed that genetic hybrids between certain strains of melanistically pigmented platyfish (*Xiphophorus maculatus*) and non-pigmented swordtails (*Xiphophorus helleri*) developed spontaneous melanomas from specialized melanin-containing cells (macromelanophores) comprising various black pigment patterns (Gordon, 1927; Haussler, 1928; Kosswig, 1927). These hybrid melanomas originate from cells within polymorphic pigment patterns derived from the platyfish strains, which become phenotypically enhanced in hybrid progeny, typically showing a large proportion of relatively undifferentiated, actively proliferating melanocytes (Anders, 1991; Gordon, 1959; Vielkind, 1976; Vielkind and Vielkind, 1982). Human melanomas also consist of melanocytes with poorly regulated proliferation (Sauter and Herlyn, 1998), and both *Xiphophorus* and human melanomas exhibit similarities in their histopathologies (Gimenez-Conti et al., 2001; Grand et al., 1941; Ishikawa et al., 1975; Sobel et al., 1975; Vielkind and Vielkind, 1970; Vielkind et al., 1971). Transplanted *Xiphophorus* melanomas are vascularized and grow in nude mice in a manner indistinguishable from transplanted human melanomas, while maintaining expression of fish antigens (Schartl and Peter, 1988).

**Figure 1:**
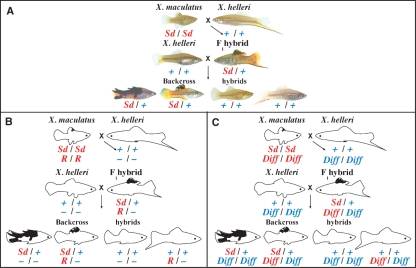
Genetics of the Gordon-Kosswig spontaneous melanoma model. (A) Hybridization of the platyfish *X . maculatus*, exhibiting the macromelanophore spotted dorsal (*Sd*) pigment pattern, to the swordtail *X. helleri* generates F_1_ hybrids with an enhanced *Sd* pigment pattern on the dorsal fin. Backcrossing F_1_ hybrids to the *X. helleri* swordtail species generates first generation backcross hybrids (BC_1_ hybrids) with three phenotypes, as shown at the bottom of panel A. Approximately one-half of the BC_1_ hybrids are non-macromelanophore pigmented fish exhibiting no melanistic pigmentation (fish shown at lower right of panel A); these hybrids have not inherited the sex-linked *Sd-Mdl* allele (designated in the figure as *Sd*) from the original platyfish parent and therefore are not susceptible to melanoma. Of the remaining approximately one-half of BC_1_ hybrids, half of these (∼ 25% of total BC_1_ progeny) are heavily pigmented and develop invasive, exophytic, nodular malignant melanoma (lower left individual in panel A) and the other half (∼ 25% of BC_1_ progeny) show enhanced *Sd* pigmentation resembling the F_1_ hybrid phenotype, but only rarely develop melanoma late in life. (B) Hypothetical two-gene inheritance model explaining the apparently Mendelian inheritance of BC_1_ phenotypes. In this model, *R* is a platyfish gene that regulates the expression of the *Xmrk* oncogene associated with the *Sd-Mdl* allele, and its total loss in heavily pigmented BC_1_ hybrids that develop melanoma explains the melanoma susceptibility of these hybrids. Heterozygosity for *R* in lightly pigmented BC_1_ hybrids results in some regulation of *Xmrk* and inhibits melanoma formation. (C) Alternative two-gene inheritance model. In this model, the autosomal locus *Diff* regulates melanoma susceptibility but is not restricted to the platyfish parent, instead existing as alleles in *Xiphophorus* spp. populations. Mendelian inheritance of melanoma susceptibility in pigmented BC_1_ hybrids is explained by homozygosity versus heterozygosity for the *X. helleri Diff* allele. These inheritance models as applied to different *Xiphophorus* crossing schemes are discussed in the text.

Early genetic analysis of melanoma formation in *Xiphophorus* hybrids was aided by the fact that pigment pattern-determining loci are sex-linked (Gordon, 1931). Another characteristic that permitted the application of classical, recombination genetics in early studies was that F_1_ hybrids are fertile, allowing the generation of interspecies backcross hybrids in genetic crossing schemes in which platyfish chromosomes were replaced by corresponding chromosomes from the swordtail species, used as the recurring backcross parent (Atz, 1962; Gordon, 1958; Kallman, 1970). Results from these studies, focused largely on pigmentation, were interpreted as genetic ‘modification’ in hybrids of the effects of the sex-linked platyfish-derived pigmentation locus by genes in the swordtail genome. It was speculated that the modifier genes could be acting either as ‘intensifiers’ contributed by the swordtail or ‘suppressors’ from the platyfish that were introduced by the initial hybridization, but then eliminated by backcrossing (as discussed in Schartl, 1995). To explain melanoma formation specifically in *Xiphophorus* backcross hybrids, Breider (1952) hypothesized that inhibitory genes from the platyfish suppress the expression of pigmentation in a species-specific manner, and their loss during backcrossing promotes melanoma formation in backcross hybrids. This interpretation represents an early articulation of the tumor suppressor gene concept, and was formalized for the *Xiphophorus* hybrid melanoma almost 25 yr later (Ahuja and Anders, 1976) based on extensive studies of a particular *Xiphophorus* hybrid melanoma model (Anders, 1967, 1991) which is represented in Figure 1.

In this genetic cross, the platyfish *Xiphophorus maculatus* is hybridized to the swordtail *Xiphophorus helleri* to generate F_1_ hybrids. Backcrossing F_1_ progeny to the *X. helleri* parental species generates first-generation backcross (BC_1_) hybrids. Although *X. maculatus* and *X. helleri* do not interbreed in natural conditions, artificial insemination can be used to produce F_1_ hybrids (Clark, 1950); natural breeding will occur between the F_1_ hybrids and the *X. helleri* recurrent backcross parent in closed colony matings. Poeciliids such as *Xiphophorus* are live-bearers, and inseminated females can store sperm for months, producing multiple broods in series of 30-day gestations. Figure 1 shows the basic elements of this crossing scheme which leads to BC_1_ hybrids with melanoma. The sex-linked melanistic pigmentation pattern ‘spotted dorsal’ (*Sd*) is exhibited as discrete, punctate black spots on the dorsal fins of *X. maculatus* individuals; the strains of *X. helleri* commonly used in this cross (*Sarabia* and *Lancetilla*) do not exhibit this pigmentation pattern and do not possess the specialized macromelanophores from which it originates. For simplicity, ‘*Sd*’ is used in the figures to represent the sex-linked genetic locus for the spotted dorsal pigment pattern. However, it should be noted that pigmentation is a complex trait involving not only the presence of macromelanophores, but their differentiation, migration, and extent of proliferation. The notation *Mdl*, for *m*acromelanophore-*d*etermining *l*ocus, has been proposed to designate the genetic loci which specify the various sex-linked macromelanophore pigmentation patterns observed in *Xiphophorus* (Wellbrock et al., 2002). As discussed below, the oncogenic *Xmrk* gene is associated with specific *Mdl* loci, but neither the pigment pattern designation (e.g., *Sd*) nor *Xmrk* should be considered as synonymous with a *Mdl*; rather, *Sd* and other pigmentation pattern-determining loci should be considered as alleles of *Mdl*. Historically, the notations *Tu* and *M* have also been used in this context (Anders, 1991; Kallman, 1975).

As represented in Figure 1(A), the F_1_ hybrid from crossing *Sd*-bearing *X. maculatus* to *X. helleri* expresses an enhanced pigmentation pattern on the dorsal fin. There is extensive melanocytic hyperplasia, or melanosis, reflecting greater proliferation of macromelanophores. Examination of cells from the enhanced pigment pattern shows altered cell morphology with a larger proportion of poorly differentiated cells, as well as more actively dividing cells than seen in pigment pattern cells from the *X. maculatus* parental strain (Ahuja et al., 1980; Siciliano et al., 1976; Vielkind, 1976; Vielkind and Vielkind, 1970); tyrosinase activity is also elevated (Vielkind and Vielkind, 1982). Backcrossing to the *X. helleri* parental strain produces BC_1_ progeny of which approximately half are non-pigmented (lower right in Figure 1A). Of the remaining half, there is roughly a 1:1 ratio of BC_1_ hybrids with enhanced *Sd* expression, resembling the F_1_ hybrid, and BC_1_ hybrids with extremely enhanced pigment patterns that may not be restricted to the dorsal fin; these heavily pigmented hybrids, about 25% of total backcross progeny, spontaneously develop exophytic, nodular, invasive melanomas. The apparently Mendelian segregation of these phenotypes is consistent with a two-gene inheritance model involving a sex-linked and an autosomal gene locus, two different interpretations of which are shown in Figure 1(B, C).

In both these inheritance models, it is assumed that the complex, sex-linked *Mdl* locus specifying the spotted dorsal pattern (*Sd*) is absent in the swordtail parent, *X. helleri*. In the model of Figure 1(B), the autosomal gene is designated as *R* for ‘regulator’ or ‘repression’ gene (Ahuja and Anders, 1976; Anders, 1967; Schartl, 1995). In this case, there are two copies of *R* in the highly inbred *X. maculatus* parental strain, resulting in tight regulation of the pigment pattern, with controlled proliferation and a large proportion of terminally differentiated macromelanophores. Hybridization to the inbred *X. helleri* parental strain, with no *R* loci, results in F_1_ hybrids heterozygous at every genetic locus; inheriting only one copy of *R* leads to some loss of regulation of the pigment pattern, resulting in *Sd* enhancement, increased proliferation, and melanosis. (This phenotype is often referred to as ‘benign melanoma,’ but we prefer ‘melanosis’ or ‘melanocytic hyperplasia’). Backcrossing F_1_ hybrids to the *X. helleri* strain generates progeny in which there is replacement of *X. maculatus* genes in F_1_ individuals based on random assortment in meiosis. These BC_1_ progeny have pigmentation phenotypes defined by the inheritance of *Sd* and *R*, as shown in Figure 1(B). BC_1_ hybrids that inherit *Sd* are pigmented, and whether or not there is some regulation of the pigment pattern (i.e., in pigmented BC_1_ hybrids that inherit *R*), or complete loss of regulation (in pigmented BC_1_ hybrids that do not inherit *R*) determines if an individual BC_1_ hybrid resembles the F_1_ phenotype or exhibits severe melanosis and is prone to developing spontaneous, primary malignant melanoma, respectively. This genetic model obviously lends itself to the interpretation that a gene associated with the complex *Sd*-*Mdl* allele behaves as a dominant oncogene, and that *R* is a classical, recessive tumor suppressor (Ahuja and Anders, 1976; Anders, 1991; Schartl, 1995).

The inheritance model represented in Figure 1(C) does not assume that the autosomal gene apparently regulating melanoma susceptibility is present only in the *X. maculatus* parent. The notation *Diff* is derived from studies of macromelanophore differentiation in *Xiphophorus* species and hybrids (Ahuja et al., 1980; Vielkind, 1976; Vielkind and Vielkind, 1982). From results of these studies, the autosomal locus *Diff* was proposed to regulate macromelanophore differentiation, modifying pigmentation phenotype to result in either its intensification or suppression in genetic hybrids. In this model, *Sd*-inheriting pigmented BC_1_ hybrids fall into one of two phenotypic categories depending on whether they are homozygous for the *X. helleri Diff* allele, or heterozygous, as represented in Figure 1(C). In the crossing scheme shown, pigmented BC_1_*X. helleri Diff* homozygotes exhibit heavy melanization and are susceptible to spontaneous melanoma (lower left individual in Figure 1C). This genetic model accommodates some early genetic observations in *Xiphophorus*, primarily studies of the inheritance of pigmentation, in which so-called ‘modifiers’ or ‘unlinked regulators’ present in the *X. helleri* genome were proposed to exert some determinative influence on the extent of pigmentation patterns and the degree of differentiated pigment cell types in hybrids (Atz, 1962; Gordon, 1958; Kallman, 1970; Zander, 1969). There is evidence supporting each of these inheritance models, as discussed in following sections.

### Molecular and biochemical characterization of the sex-linked *Xmrk* oncogene

Early molecular studies were aimed at identifying the sex-linked oncogene, then called *Tu* (for ‘tumor’), associated with the *Sd* pigmentation pattern-determining locus in *X. maculatus.* The initial focus was on receptor tyrosine kinases (RTKs), since high levels of expression of SRC*,* FYN*,* YES and a Rous sarcoma virus-related kinase were observed in *Xiphophorus* hybrid melanomas (Barnekow et al., 1982; Hannig et al., 1991; Schartl et al., 1982, 1985). Co-segregation of an epidermal growth factor receptor (*EGFR*)-related restriction fragment length polymorphism with *Tu* was reported in 1988 (Adam et al., 1988), and Schartl and co-workers subsequently isolated a novel receptor tyrosine kinase sequence related to *EGFR* which satisfied the genetic criteria for *Tu,* naming it *Xmrk* for *Xiphophorus m*elanoma *r*eceptor *k*inase (Wittbrodt et al., 1989). In addition to its isolation by positional cloning, *Xmrk* was confirmed to be the *Tu* oncogene by the demonstration that deletion or disruption of this gene abrogated the potential to cause melanomas in hybrid fish (Schartl et al., 1999). *Xmrk* was observed to be overexpressed in melanomas arising in *Xiphophorus* BC_1_ hybrids (Adam et al., 1991) and also in melanomas occurring in certain pigmented, non-hybrid fish (Kazianis and Borowsky, 1995; Schartl et al., 1995). A very recent publication (Schartl et al., 2010) demonstrates that a *Xmrk* transgene in medaka is capable of inducing melanomas; this study is discussed in some detail later in this review. All these observations support the conclusion that *Xmrk* can function as a dominant, melanoma-inducing oncogene.

Initial molecular analysis of the *Tu* region of the sex chromosome revealed that *Xmrk* had resulted from a gene duplication and a subsequent rearrangement that fused an adventitiously acquired promoter to the melanoma-inducing copy of *Xmrk* (Adam et al., 1992). These findings resulted in the designations ONC-*Xmrk* (for the oncogenic copy) and INV-*Xmrk* (for the original, ‘invariant’ copy) to distinguish *Tu* from the original *EGFR*-related gene (other workers have referred to these duplicated genes as *Xmrk-2* and *Xmrk-1,* respectively; see Kazianis et al., 2000; Woolcock et al., 1994). However, subsequent work by Schartl and colleagues (Gomez et al., 2004) established that INV-*Xmrk* is an ortholog of *EGFR*, which is genomically duplicated in *Xiphophorus,* and the designations *egfrb* and *Xmrk* are now preferred for the original and oncogenic sex-linked gene copies. Recently, bacterial artificial chromosome (BAC) contigs have been assembled for the subtelomeric sex-determining region of *X. maculatus* sex chromosomes (Schultheis et al., 2006), revealing that the sex determination region in *X. maculatus* is unstable and subject to frequent duplications, deletions, and transpositions. As suggested by these authors, this instability may help to explain the highly polymorphic nature of macromelanophore pigment patterns and melanoma phenotypes, as well as other sex-linked polymorphic traits in *Xiphophorus* such as age at onset of sexual maturity.

Molecular analysis of *Xmrk* and *egfrb* gene structure and expression has shown that both loci are closely linked to the *Mdl* locus, mapping within 0.6 cM on the *X. maculatus* X chromosome, physically located ≥1 Mb apart with *Xmrk* closer to the telomere (Gutbrod and Schartl, 1999; Schultheis et al., 2006). Each gene is about 23 kb in size, with exon-intron structures very similar to the receptor tyrosine kinases found in higher vertebrates (Gutbrod and Schartl, 1999). Transcript sizes are polymorphic, with *egfrb* producing a 5.8 kb transcript and *Xmrk* producing a 4.7 kb transcript (Adam et al., 1991). The *egfrb* gene is ubiquitously expressed at low levels (Dimitrijevic et al., 1998) and is developmentally modulated during embryogenesis and organogenesis (Wittbrodt et al., 1989). The *Xmrk* transcript, by contrast, is not detected in any tissues except pigment cells in the pigment patterns giving rise to melanomas in hybrids, and in the melanomas, being highly expressed in both contexts (Dimitrijevic et al., 1998; Kazianis et al., 2000; Wellbrock et al., 1998; Woolcock et al., 1994). Molecular characterization of the regions upstream from both genes has shown that the promoters are completely different from each other. The *egfrb* promoter has features of a housekeeping gene, consistent with its observed expression patterns. The *Xmrk* promoter is apparently derived from a unique *D* (for ‘donor’) locus on the sex chromosome, which is amplified and distributed throughout the *Xiphophorus* genome. The *D* locus is associated with a zinc finger gene and a gene of unknown function in the amplified regions (Fornzler et al., 1996; Nanda et al., 1996; Schartl et al., 1994). A recombination event involving this amplified structure is hypothesized to have created a novel promoter for *Xmrk* (Fornzler et al., 1996; Nanda et al., 1996). Various transcription factor motifs have been identified in the *Xmrk* promoter, including a GC box (Baudler et al., 1997) and a CpG island. The CpG island is hypermethylated in non-hybrid fish but hypomethylated in melanized tissues from hybrids and in a melanoma-derived fish cell line, which may contribute to *Xmrk* expression characteristics during melanomagenesis (Altschmied et al., 1997).

**Figure 2:**
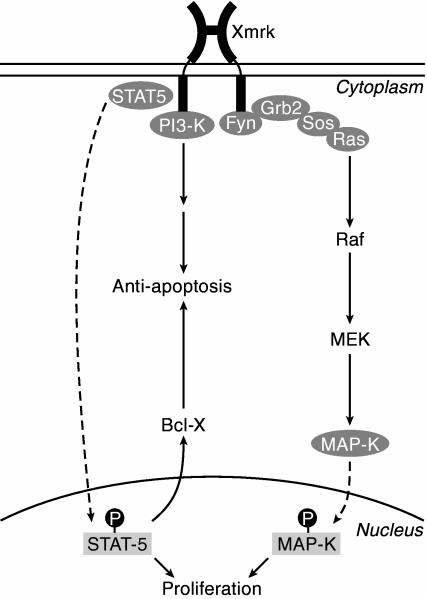
Simple model for activation of downstream proliferation and pro-survival pathways by *Xmrk*. The *Xmrk* oncogene is constitutively activated in melanocytes constituting *Xiphophorus* macromelanophore pigment patterns. Signaling through STAT5 and PI3K pathways evokes both proliferation and anti-apoptosis, as shown at the left of the figure. *Xmrk* also orchestrates downstream RTK signaling mediated by FYN and the RAS-RAF-MEK-ERK cascade leading to phosphorylation of MAP kinase and its activation, providing further proliferation stimulus as shown at the right. Activation of *Xmrk* has multiple other downstream effects, as extensively discussed in Meierjohann and Schartl (2006).

As noted above, *Xmrk* is highly expressed in pigment cells, and significantly more *Xmrk* overexpression in melanomas is a critical feature of malignant transformation by this oncogene. However, comparing the *Xmrk* oncoprotein to its *Egfrb* progenitor also reveals amino acid residue differences that contribute to its oncogenicity as well as specific mutations in *Xmrk* that lead to its dimerization and constitutive activation in melanoma cells (Dimitrijevic et al., 1998; Meierjohann et al., 2006a; Winnemoeller et al., 2005). A large body of biochemical studies from Schartl’s group, using fish and cell culture models, has characterized cellular responses to *Xmrk* activation (reviewed in Meierjohann and Schartl, 2006). *Xmrk*-initiated signaling mimics binding of a growth factor ligand to transmembrane receptor (Gomez et al., 2001), and activates multiple downstream signaling cascades through the RAS-RAF-MEK-ERK (MAPK) and PI3K-AKT pathways, as well as activating STAT5, resulting in robust proliferation signaling and survival (anti-apoptotic) responses (Baudler et al., 1999; Hassel et al., 2008; Morcinek et al., 2002; Wellbrock and Schartl, 1999; Wellbrock et al., 1995, 1999). Figure 2 represents a simplified model for some of the downstream effects of *Xmrk* activation in melanoma cells; a more complete and detailed model is provided in Meierjohann and Schartl (2006). In addition to initiating the signaling cascades illustrated in Figure 2, *Xmrk* has been shown to induce motility in melanocytes through its interaction with FYN to stimulate the focal adhesion kinase, which modulates focal adhesions (Meierjohann et al., 2006b). There are also additional effects downstream from phosphorylated MAPK that facilitate survival responses and inhibit melanocyte differentiation (Meierjohann and Schartl, 2006). The many physiological responses downstream from *Xmrk* in pigment cells and melanomas derived from them in *Xiphophorus* are thus consistent with robust, cell type-specific proliferation and anti-apoptosis, characteristic of these tumors.

*Xmrk* is a potent oncogene, and yet is maintained in wild populations, leading to speculation that it has been retained during evolution by conferring some selective advantage(s). One hypothesis is that *Xmrk* could be acting as a speciation gene, shielding a species from hybridization by being deleterious in genetic hybrids; however, there are a number of arguments against this notion as applied to *Xmrk* (Schartl, 2008). Nonetheless, a recent, intriguing study of mating behavior in *Xiphophorus cortezi* showed that females prefer males with enhanced spotted caudal (*Sc*) pigmentation patterns (Fernandez and Morris, 2008). All *Xiphophorus* species with *Xmrk* exhibit a macromelanophore pigment pattern of some type (Weis and Schartl, 1998). The *Sc* pattern was confirmed to be associated with *Xmrk* in this study, and it was concluded that sexual selection was responsible for maintaining the *Xmrk* oncogene in *X. cortezi* populations. For the individual male, the deleterious effect of *Sc* enhancement, increased melanoma risk, is counterbalanced by increased male acquisition of females. One of the wild populations studied had a higher frequency of *Sc* among females, mitigating the mating preference displayed in the other populations and suggesting that sexual selection for *Sc* was frequency dependent. As the authors point out, these results are relevant to the evolutionary origin of cancer, since recent findings have demonstrated that several types of cancer are under positive selection (Crespi and Summers, 2006). The *Xiphophorus* hybrid melanoma models thus offer an excellent experimental avenue for further investigation of the molecular genetics underlying this observation.

### Genetic characterization of the autosomal melanoma susceptibility locus

Although *Xmrk* behaves as a frank oncogene in many contexts, including in some non-hybrid *Xiphophorus* species with differing macromelanophore pigment patterns (Fernandez and Morris, 2008; Kazianis and Borowsky, 1995; Schartl et al., 1995), an additional, autosomal gene is required to explain melanoma formation in most *Xiphophorus* hybrid models. Early efforts aimed at identifying this gene were focused on gene mapping approaches. Because interspecies genetic hybrids are extremely polymorphic, separation of proteins based on charge differences by isozyme electrophoresis in starch gels (which allows activity staining) provides a powerful genotyping methodology that enables mapping of gene linkages by analyzing genetic recombination in BC_1_ hybrids (Morizot et al., 2001). Construction of a genetic linkage map for *Xiphophorus* was initiated using this approach in the 1970s, and a locus called *Mel Sev* (for ‘melanoma severity’) was linked to esterase-1 in *Xiphophorus* by Siciliano et al. (1976). This linkage assignment for the *Diff* autosomal melanoma susceptibility locus was confirmed by later studies (Ahuja et al., 1980; Morizot and Siciliano, 1983). The development and application of DNA polymorphisms, including microsatellites, for linkage mapping in *Xiphophorus* has resulted in more robust gene maps and a more detailed, finer scale map of the LG V region encompassing the *Diff* locus (Kazianis et al., 2004b; Walter et al., 2004).

The search for *Diff* candidate genes initially relied on cloning and mapping of the *Xiphophorus* homologs of likely tumor suppressors. As a result, *p53*, *RB* and *CDKN2* homologs, as well as other genes involved in tumorigenesis, were isolated from *Xiphophorus* using various cloning strategies and mapped (Butler et al., 2007; Kazianis et al., 1998a; Morizot et al., 1998; Nairn et al., 1996a). However, mapping of these sequences did not show linkage to any LG V markers until genetic analysis of a UV-inducible *Xiphophorus* melanoma model (described in the next sections) revealed significant linkage of the heavily melanized pigmentation phenotype and melanoma susceptibility to a *CDKN2*-related sequence that mapped to LG V (Nairn et al., 1996b). This gene, named *CDKN2X*, localized to the region of LG V expected to contain the *Diff* locus, and was also an attractive candidate for *Diff* because of the well established association of *CDKN2A* mutations with susceptibility to melanoma in humans (Chin et al., 2006). However, subsequent studies (Kazianis et al., 1999, 2000) revealed that *CDKN2X* was hypomethylated and overexpressed in melanized skin and melanomas, an unexpected characteristic based on the role of *CDKN2A* in human melanoma as currently understood (Ruas and Peters, 1998), although *CDKN2A* is overexpressed in some other human cancers. For example, *CDKN2A* is overexpressed in an experimental mouse bladder carcinoma (Asamoto et al., 1998), in some human breast cancers (Emig et al., 1998), and in ovarian carcinomas, in which overexpression may be an early event in tumorigenesis (Shigemasa et al., 1997).

Molecular cloning and analysis of the *CDKN2X* alleles from *X. maculatus* and *X. helleri* revealed only two amino acid differences (Kazianis et al., 1999; Nairn et al., 2001), which are not predicted to affect their activities as CDK inhibitors based on functional studies of mammalian CDKN2 proteins. A noteworthy structural difference between *CDKN2X* and mammalian *CDKN2A* genes is the absence of the alternative reading frame encoding the ARF protein in *CDKN2A*. In addition, there is no evidence in *Xiphophorus* of the *CDKN2A/CDKN2B* gene duplication existing in mammals. In *Fugu*, a Tetraodontiform fish with a compact genome, it has been established that a corresponding gene called *INK4AB* also is not associated with an *ARF* or gene duplication, and that only a single additional paralog (*INK4D*) likely exists in the *Fugu* genome (Gilley and Fried, 2001). A *CDKN2D* gene was cloned from *Xiphophorus* and analysis of its structure in comparison to *Fugu INK4D*, *Xiphophorus CDKN2X* and the mammalian *CDKN2* family revealed that the same was true, leading to re-naming the *CDKN2X* gene *CDKN2AB* (Kazianis et al., 2004a). Thus, in both Tetraodontiform and Cyprinodontiform fishes this gene appears to be ancestral to both *CDKN2A* (*INK4A*) and *CDKN2B* (*INK4B*), and both duplication and association with *ARF* of this ancestral gene occurred after the evolutionary divergence of the lineage leading to mammals from fish.

**Figure 3:**
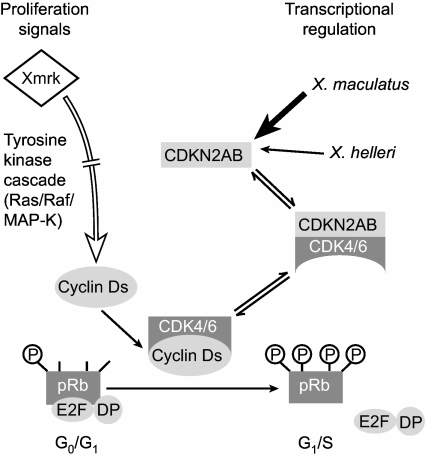
Hypothetical model for the possible role of CDKN2AB in regulating proliferation at the G1/S checkpoint. In the generalized model depicted, hyperphosphorylation of the retinoblastoma protein (pRb) releases transcription factor E2F and its dimerization partner (DP), which represent members of a family of transcription factors that upregulate many genes necessary for DNA synthesis. This step is controlled by the cyclin-dependent kinase inhibitor (CDKN2) family in mammalian cells, which bind to CDK4 and CDK6 and prevents their binding to Cyclin Ds (or E); this step may be similarly regulated by CDKN2AB in *Xiphophorus* melanocytes. In a situation where persistent and strong proliferation signals are generated, (shown at top left) originating from overexpression of *Xmrk* in melanoma cells through tyrosine kinase-mediated signaling pathways, there may be compensation by CDKN2AB overexpression (top right). For a UVB inducible melanoma model (shown in Figure 4D), Kazianis et al. (1999) have shown that in *CDKN2AB* heterozygotes with melanomas, there is marked differential expression of this proliferation inhibitor in melanoma tissue, with the *X. maculatus CDKN2AB* allele overexpressed >11-fold compared to the *X. helleri* allele, suggesting the possibility that greater expression of *X. maculatus CDKN2AB* (thick filled arrow, upper right) relative to the expression levels capable from *X. helleri CDKN2AB* (thin filled arrow, upper right) might partially compensate in heterozygotes for the strong proliferation signals driven by *Xmrk* overexpression. *Used with permission.*

Although the p13 (13 kDa) proteins encoded by the *X. maculatus* and *X. helleri CDKN2AB* alleles are almost identical, numerous sequence differences are evident in the promoter regions of these two alleles, leading to speculation that differences in transcriptional regulation could be involved in expression of the melanoma phenotype in BC_1_ hybrids (Butler et al., 2007; Nairn et al., 2001). In rare *CDKN2AB* heterozygous BC_1_ hybrid melanomas there is significant *CDKN2AB* overexpression, as also seen in homozygous BC_1_ hybrid melanomas; however, allele-specific RT-PCR analysis of gene expression shows significant differential overexpression of the *X. maculatus CDKN2AB* allele relative to the *X. helleri* allele (>11-fold) in melanomas from these heterozygotes (Kazianis et al., 2000). A model for the possible role of *CDKN2AB* in melanoma formation was proposed (Nairn et al., 2001) in which the differential expression of the *X. maculatus* and *X. helleri* alleles in *Xmrk*-inheriting BC_1_ hybrids results in dysregulation of the G1/S checkpoint and loss of control of melanocytic proliferation, leading to melanoma formation (Figure 3). In this model, the pigmented *X. maculatus* parental strain robustly expresses CDKN2AB (heavy arrow) mitigating the effects of *Xmrk* overexpression and resulting in controlled melanocytic proliferation and small, discrete pigment spots. By contrast, in F_1_ hybrids and the fraction of BC_1_ hybrids that are *CDKNAB* heterozygotes, there is some loss of regulation of *Xmrk* activity due to less overall *CDKNAB* expression from the two different alleles (postulating that the *X. helleri* allele is a weak expressor, thin arrow) resulting in pigment pattern enhancement and benign hyperplasia. According to this model, in the BC_1_ hybrids that are homozygous for the *X. helleri CDKN2AB* allele, there is virtually complete loss of control of melanocyte proliferation in the macromelanophore pigment patterns and melanomas are formed.

**Figure 4:**
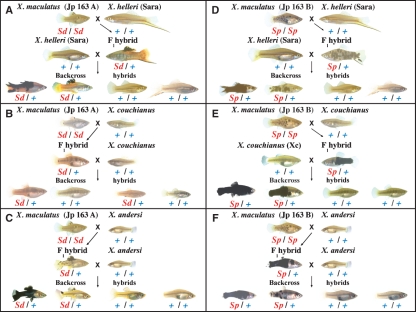
Crossing schemes for generating backcross hybrids. (A) Gordon-Kosswig spontaneous melanoma model (also shown in Figure 1): In this cross, *X. maculatus* Jp 163 A, carrying the spotted dorsal (*Sd*) pigment pattern locus, is mated to *X. helleri,* which is wild-type (+/+) for this macromelanophore pigment pattern locus. F_1_ hybrids are then crossed back to *X. helleri,* and the first backcross generation exhibits heavy (*Sd*/+) and light (+/+) pigmentation phenotypes. In this crossing scheme, segregation of the *Diff* locus determines heavy and light pigmentation classes in the first backcross generation of the pigmented backcross progeny (i.e. the one-half of total backcross progeny inheriting *Sd* from the *X. maculatus* Jp 163 A parent) the heavily pigmented backcross hybrids (lower left) are homozygous for the *X. helleri Diff* locus, whereas the lightly pigmented hybrids (second from lower left) are heterozygous for *Diff*, as is the F_1_ hybrid. Melanomas develop spontaneously in the homozygous, heavily pigmented backcross hybrids; (B) Spotted dorsal –*X. couchianus* (Sd-*couchianus*) cross: In this cross, instead of *X. helleri* being used as the backcross parent as in (A), a platyfish species, *X. couchianus* is used. Even though it can be demonstrated genetically that one-half of the backcross progeny inherit the sex-linked *Sd* locus, there is suppression of the expression of this pigment pattern locus in both the F_1_ and backcross hybrids. (C) Spotted dorsal –*X. andersi* (Sd-*andersi*) cross: In this cross, *X. maculatus* Jp 163 A and the platyfish species *X. andersi* are used. There is overexpression of the *Sd* pigment pattern in F_1_ hybrids, and a wide range of pigmentation phenotypes is observed among pigmented backcross hybrids. Pigmentation enhancement in hybrids is non-*Diff* regulated in this cross (Vielkind et al., 1989). (D) Spotted side –*X. helleri* (Sp-*helleri*) UV-inducible melanoma model: This cross is the same as in (A), except *X. maculatus* Jp 163 B, carrying the spotted side (*Sp*) pigment pattern locus, is used. Melanomas can be induced by UV in both the heavy and light classes (see Nairn et al., 1996b; Setlow et al., 1989; and text). (E) Spotted side –*X. couchianus* (Sp-*couchianus*) UV-inducible melanoma model: In this cross, *X. maculatus* Jp 163 B is mated to *X. couchianus*, as for the cross shown in (B). Instead of suppression of pigment pattern expression, there is dramatic enhancement of the spotted side pigment pattern in F_1_ hybrids. F_1_ hybrids are then crossed back to *X. couchianus,* and the *Sp*-inheriting backcross hybrids exhibit heavy and light pigmentation phenotypes as shown. Melanomas have been reported to be induced in both classes by UVB and UVA wavelengths (Setlow et al., 1993). (F) Spotted side –*X. andersi* (Sp-*andersi*) hybrid cross: In this cross, *X. maculatus* Jp 163 B is mated to *X. andersi* and F_1_ hybrids are crossed back to *X. andersi*, as in (C). These animals exhibit a wide range of light and heavy pigmentation phenotypes among *Sp*-inheriting backcross progeny, and pigment pattern enhancement and spontaneous melanoma susceptibility are non-*Diff* controlled, as for the cross shown in (C). BC_1_ hybrids are refractory to UVB induction of melanomas (see text). *Used with permission.*

*Xmrk* activates the RAS-RAF-MEK-ERK (MAPK) pathway, resulting in strong proliferation signaling (Figure 2). In human melanoma development, benign nevi often harbor *BRAF* mutations (e.g., V600E), which convert it into an active oncogene. Paradoxically, such nevi persist in a growth arrested state, rarely progressing to melanomas (Bennett, 2003). However, this can be at least partly explained by the fact that oncogenic BRAF induces *CDKN2A* expression, promoting oncogene-driven senescence and maintaining the nevi in a benign state (Michaloglou et al., 2005). In human melanoma development, transformation from this benign state of cell senescence to malignancy may require additional genetic or epigenetic changes which enable *BRAF* to exert its oncogenic effect. As suggested by Butler et al. (2007), *Xiphophorus* hybrid melanomas may represent a somewhat parallel situation in which regulation of *CDKN2AB* expression in melanocytes in pigment patterns determines whether their proliferation is controlled or uncontrolled. Rather than additional mutational changes, the substitution of different *CDKN2AB* alleles with differing expression characteristics in hybrid genetic backgrounds may influence the degree to which the G1/S checkpoint can be ‘balanced’ through its maintenance by cyclin-dependent regulation of Rb phosphorylation, regulated in turn by *CDKN2AB* (Figure 3). Since *Xmrk* is such a potent oncogene, in *Xiphophorus* hybrid melanomas mutational changes in other genes may not be necessary for melanoma development once this equilibrium is disrupted.

To further investigate differences in transcriptional regulation of the *X. maculatus* and *X. helleri CDKN2AB* alleles, reporter genes were constructed containing upstream regions of these alleles and expression analysis was performed in *Xiphophorus* cell lines of melanoma (PSM cells, Wakamatsu, 1981) and non-melanoma (A2 cells, Kuhn et al., 1979) origin; these two *Xiphophorus* cell lines have been extensively used by Schartl and colleagues to study *Xmrk* biochemistry (Wellbrock et al., 2002). Results of deletion analysis suggested that there is a series of positive- and negative-acting elements present in the *X. maculatus CDKN2AB* promoter which are absent or less active in the *X. helleri* promoter (A. Butler, M. Friedersdorf, D. Trono, P. de Forest, J. Plummer, J. Rahn, R.S.N, submitted). Of particular interest, experimental results from this study showed that a perfect Sp consensus sequence in the untranslated region (UTR) of *X. helleri CDKN2AB* is mutated in *X. maculatus* such that it has lost its responsiveness to Sp3, which is a negative transcriptional regulator abundant in fish cells. It is possible that the mutated Sp consensus sequence in the *X. maculatus CDKN2AB* UTR may have been selected to lose its response to Sp3 in co-evolution with *Xmrk*, resulting in loss of negative transcriptional regulation of *CDKN2AB* in this pigmented species and more robust expression. This might contribute to explaining how the strong proliferative effects of *Xmrk* are mitigated in *Xiphophorus* species that exhibit macromelanophore pigment patterns, and become uncontrolled in some hybrids. Further studies of a number of different species and different *Xiphophorus* melanoma models will be required to elucidate this issue.

It must be noted that the genetic evidence supporting *CDKN2AB* as a candidate gene for *Diff* is stronger that the functional evidence. A study by Kazianis et al. (1998b) used over 1100 BC_1_ hybrid fish from four different crossing schemes which involved both *Sd* and *Sp* pigment patterns to show an robust association of *CDKN2AB* with *Diff* (as defined by zygosity-controlled pigmentation phenotype). In this study, quantitative trait linkage (QTL) analysis revealed a significant likelihood ratio statistic (>10) generated from a whole-genome permutation test. However, the fact remains that *CDKN2AB* is overexpressed in *Xiphophorus* melanomas, which is not consistent with the behavior predicted from the role of *CDKN2A* in human melanomagenesis (Ruas and Peters, 1998), or the function of a classical tumor suppressor gene, as illustrated for *R* in Figure 1(B). This oncogene-tumor suppressor gene model, in which *R* is exclusively a platyfish gene and acts upstream to regulate *Xmrk* expression or activity, and loss of both copies of *R* results in melanoma-susceptible BC_1_ hybrids, is ascendant in current thinking about the molecular genetic basis of spontaneous melanoma formation in the ‘classical’ Gordon-Kosswig *Xiphophorus* model shown in Figure 1(A) (Meierjohann and Schartl, 2006). However, the inheritance model represented in Figure 1(C), in which *Diff* as the autosomal determinant of melanoma susceptibility exists as alleles in different *Xiphophorus* species is conceptually broader, and can explain the inheritance of pigmentation and melanoma susceptibility phenotypes in the Gordon-Kosswig cross equally as well as the model of Figure 1(B). As discussed in the next section, it also can explain other hybrid phenotypes showing suppression or differing degrees of pigment pattern enhancement and different susceptibilities to melanoma formation, as observed in *Xiphophorus* BC_1_ hybrids generated from different genetic crossing schemes.

### Inducible melanoma formation and photocarcinogenesis in *Xiphophorus* hybrid melanoma models

The preceding discussion has largely been concerned with the roles of *Xmrk* and *Diff* in the so-called ‘classical’ or Gordon-Kosswig *Xiphophorus* hybrid melanoma model shown in Figure 1. However, as discussed in this section, numerous genetic crossing schemes have been developed using different *Xiphophorus* species, primarily for purposes of studying the inheritance of other pigmentation patterns and sex-linked characteristics such as fecundity and age at onset of sexual maturity. Some of these crosses also provide additional melanoma models. BC_1_ progeny from some crosses exhibit lower spontaneous melanoma frequencies than those from the classical *Xiphophorus* hybrid melanoma model. One genetic cross, in fact, shows complete suppression of pigmentation in BC_1_ hybrids that inherit the *Sd-Mdl* allele from the same *X. maculatus* parental strain used in the Gordon-Kosswig cross (Figure 4B). Also shown in Figure 4, BC_1_ hybrids from different crosses can exhibit different degrees of pigment pattern enhancement, depending on the specific *Mdl* allele and, importantly, on what species of non-melanin pigmented *Xiphophorus* is used as the recurrent backcross parent.

For example, panels A–C of Figure 4 represent three different genetic crosses all of which use the platyfish *X. maculatus* strain Jp 163 A as the pigmented parent. However, three different *Xiphophorus* species are used as the recurrent backcross parent. Panel A shows the Gordon-Kosswig cross also shown in Figure 1, in which the *Sd-Mdl* allele (designated as ‘*Sd*’) conferring the spotted dorsal pattern is enhanced in F_1_ hybrids and which generates spontaneous melanoma-susceptible BC_1_ hybrids after backcrossing to the swordtail parent *X. helleri.* However, if the platyfish *X. couchianus* is substituted for *X. helleri* in this crossing scheme (panel B), there is complete suppression of the *Sd-Mdl* allele, and neither F_1_ nor BC_1_ hybrids are pigmented. If the same crossing scheme is used, but the platyfish *X. andersi* is substituted as the recurrent backcross parent (panel C) there is enhancement of *Sd* in F_1_ hybrids and a wide range of pigmented phenotypes in BC_1_ hybrids, from very light (resembling the *X. maculatus* parent) to very heavy. Crosses represented in panels D–F show the effects on pigmentation phenotype of substituting *X. maculatus* strain Jp 163 B containing the spotted side *Sp-Mdl* allele (*‘Sp’*) for *X. maculatus* Jp 163 A in these same crossing schemes. Of particular interest is comparison of panels B and E, showing that the *Sp* phenotype is extremely enhanced in F_1_ and BC_1_ hybrids in the *X. couchianus* hybrid background (panel E), compared to complete suppression of *Sd* in the same genetic background (panel B). It should be noted that *X. maculatus* strains Jp 163 A and Jp 163 B derive from a single female collected from the wild (Rio Jamapa) that exhibited both *Sd* and *Sp* pigment patterns, which were separated after nine generations of brother-sister matings into these two pigmented strains (Kallman, 1975). These two strains are highly inbred (>90 generations of closed colony matings) and thus are very closely related genetically.

These observed phenotypes are not easily explained by the inheritance model of Figure 1(B). A more accommodating inheritance model is shown in Figure 1(C), in which some gene(s) inherited from the recurrent backcross parent modify the effects of *Xmrk* activity in pigmented BC_1_ hybrids. This model explains the pigmentation phenotypes observed in the Gordon-Kosswig model and also can explain other phenotypes in which there is suppression or differing degrees of pigment pattern enhancement. In addition, it can account for the different susceptibilities to spontaneous melanoma formation observed in BC_1_ hybrids generated from a variety of genetic crosses. Any highly inbred strain used as the recurrent backcross parent possesses its own *Diff* allele, thus BC_1_ hybrids homozygous for this allele would exhibit the most pronounced effect, and *Diff* heterozygotes a less pronounced effect, as the result of modifying the activity of a particular *Mdl* in the hybrid genetic background. Other autosomal loci may also play important roles. For example, macromelanophore pigmentation in the crosses shown in Figure 4(C, E) are not regulated by the LG V *Diff* gene, but by another, non-LG V autosomal locus (Vielkind et al., 1989). These genetic complexities are difficult to reconcile with the simple oncogene-tumor suppressor gene model in which *R* is contributed by the pigmented *X. maculatus* parent and lost through backcrossing. The application of QTL analysis for identifying other genes that may be involved in melanoma formation and progression in these models offers an attractive experimental avenue for further genetic studies of the *Xiphophorus* melanoma models; this approach is discussed in more detail later in this review.

Pigmented BC_1_ hybrids from the crosses represented in Figure 4 also display different melanoma susceptibilities. Except for the Gordon-Kosswig hybrid model (Figures 1 and 4A), none of the BC_1_ hybrids shown exhibit a particularly high incidence of spontaneous melanoma formation. This characteristic has been exploited by several investigators to study induced melanoma in some of these models. Notably, Setlow and colleagues first used the hybrid model shown in Figure 4(D) (called Sp-*helleri* to designate the *Sp-Mdl* allele backcrossed into the *X. helleri* genetic background) to study UVB induction of melanoma, demonstrating several-fold increased induction of melanoma at 4–6 months after irradiation of 5-day-old fry (Setlow et al., 1989). Nairn et al. (1996b) later confirmed UVB induction of melanoma in this model, and performed genetic analysis showing that induced melanoma susceptibility in the Sp-*helleri* model was linked to the LG V *Diff* locus, and paralleled the genetics of the Gordon-Kosswig (i.e., Sd-*helleri*) cross. Setlow’s group also used the cross shown in Figure 4(E) (Sp-*couchianus*) to investigate the UV wavelength dependence of melanoma (Setlow et al., 1993). The action spectrum for melanoma published in this study has significantly contributed to a prolonged controversy over the relative importance of UVA and UVB wavelengths in inciting melanoma in the human population (Lund and Timmins, 2007; Mitchell and Nairn, 2006). Studies designed to resolve this controversy using *Xiphophorus* and other experimental melanoma models are currently in progress (Mitchell et al., 2007).

The direct acting mutagen *N*-methyl-*N*-nitrosourea (MNU) has also been used to induce melanoma in some of the crosses shown in Figure 4, including Sp-*helleri* (panel D) and Sp-*andersi* (panel F). The frequency of MNU-induced melanoma in pigmented BC_1_ hybrids was higher than for UVB in the Sp-*helleri* model (Kazianis et al., 2001) and no association of melanoma susceptibility with the *Diff* locus was found, in contrast to UVB induced melanomas in this model (Nairn et al., 1996b). In the Sp-*andersi* model, in which pigmentation is non-*Diff* regulated (Vielkind et al., 1989), MNU treatment also induced melanomas in pigmented BC_1_ hybrids at a significant frequency (29.7%), whereas UVB failed to induce melanomas above the background incidence (<3%). These results suggest that there may be different mechanisms for melanoma induction by MNU and UV. A recent study (Rahn et al., 2009) tested the hypothesis that MNU could be directly inactivating *CDKN2AB* by mutation. MNU-induced melanomas from F_1_ and BC_1_*CDKN2AB* heterozygotes were excised and the *X. maculatus CDKN2AB* alleles from isolated DNA samples were sequenced. However, no mutations were found, suggesting that *CDKN2AB* inactivation was not a mechanism for MNU-induced melanomagenesis in this model.

*Xiphophorus* hybrid melanoma models have thus been very useful for establishing the importance of RTK signaling pathways in melanoma formation, and providing experimental models in which genetic components can be isolated in different hybrids. From the perspective of comparative pathobiology, although mutationally altered *EGFR* is not the primary culprit in human melanoma, the *Xmrk* oncogene is upstream from and orchestrates many of the same signaling cascades known to be activated in human melanoma (Chin et al., 2006), such as the MAPK pathway. In fact, constitutive activation of this pathway in *Xiphophorus* melanoma was recognized very early as critical to melanoma causation in this model; other oncogenic and pro-survival effects of *Xmrk*, mediated through STAT5 and other effectors (e.g., PI3K) also recapitulate some of the MAPK-independent pathways important in human melanoma (see Meierjohann and Schartl, 2006, for discussion of this point). On the other hand, from genetic analysis the *CDKN2AB* homolog appears to be involved as an autosomal genetic determinant of melanoma in some of the *Xiphophorus* melanoma models, but since it is overexpressed, *CDKN2AB* does not appear to play a role in *Xiphophorus* melanoma analogous to the *CDKN2A/ARF* locus in human melanoma. Functional studies have shown that the two *CDKN2AB* alleles from *X. helleri* and *X. maculatus* are regulated differently at the transcriptional level, consistent with observations of their allele-specific expression in *Xiphophorus* melanomas (Kazianis et al., 2000). Studies in fish and human cell lines also show that the p13 proteins from the two different species each interact with CDK4/6 and inhibit CDK4/6-dependent phosphorylation of pRB, consistent with a role in regulating the G1/S checkpoint (A. Butler and R.S.N, unpublished). More functional studies will be necessary to fully clarify the role of *CDKN2AB* in melanoma susceptibility. However, it is possible that other candidate genes for *Diff,* as well as other autosomal genetic determinants of *Xiphophorus* melanoma, will be revealed by further genetic analysis. Finally, it should be noted that investigation of *Xiphophorus* melanoma models has contributed significantly to the field of photocarcinogenesis and the role of sunlight in melanoma causation (Mitchell and Nairn, 2006; Mitchell et al., 2007; Setlow, 1999; Setlow and Woodhead, 1994). Resolving some of the controversies surrounding the issue of the UV wavelength dependence of melanoma induction will depend on further studies using *Xiphophorus* in addition to other animal melanoma models, as discussed later in this review.

## Transgenic models of melanoma in fish

### Zebrafish as a model for cancer

In the 1960–1970s, George Streisinger had the vision to generate an experimental model system that could be as easily maintained and as genetically tractable as the fruit fly and worm – with the key difference of having a vertebrate body plan (Grunwald and Eisen, 2002). From this pioneering work, in the 1980s and 1990s, *Danio rerio*, commonly called the zebrafish, emerged as an important developmental and genetic vertebrate system (Patton and Zon, 2001). Like other vertebrates, zebrafish develop benign and malignant cancers, with similar histological, molecular and pathological features to human cancers (Amatruda et al., 2002). Zebrafish rarely develop spontaneous cancer, but can develop tumors in almost all tissue types after water borne treatment with carcinogens (Spitsbergen et al., 2000a,b;). Histopathological analysis indicates that zebrafish tumors share many salient features with the cancers derived from the analogous tissue in humans (Amatruda and Patton, 2008; Amatruda et al., 2002; Spitsbergen et al., 2000a,b; Stern and Zon, 2003). The shared histopathological features between zebrafish and human cancers are further mirrored in their shared molecular features. For example, the molecular signatures of the progressive stages of liver neoplasia – from adenoma to carcinoma – are shared between zebrafish and human liver cancer (Lam et al., 2006).

Like *Xiphophorus*, large numbers of zebrafish can be grown within the laboratory at relatively low cost, and development from a single-cell embryo to adulthood takes about 3 months. Importantly, however, unlike *Xiphophorus*, zebrafish fertilize their eggs externally, and hundreds of single-cell embryos can be collected each week from a pair of fish. The embryos are transparent, and key aspects of embryogenesis – from the first cell division to gastrulation cell movements and organogenesis to melanocyte pigmentation – can be viewed under the light microscope. The zebrafish genome is sequenced, and is only one of three vertebrate species, including human and mouse, that has comprehensive coverage of the genome (http://www.ensembl.org/Danio_rerio/Info/Index; D. Stemple, personal communication). Importantly, cancer and tumor suppressor genes are conserved between fish and people, as are key signaling, DNA damage, apoptosis, and senescence pathways (Amatruda and Patton, 2008).

Anticipating the value of such a system for large-scale genetic screens, as well as cancer genetics, Streisinger laid the groundwork for genetic screens in the zebrafish system (Grunwald and Eisen, 2002; Streisinger et al., 1981). Large-scale genetic screens in zebrafish have since identified hundreds of mutations that cause specific developmental and adult phenotypes providing an unprecedented window into vertebrate development. Some genetic screens have also identified new pathways and known cancer genes that promote or modify the occurrence of tumorigenesis in the adult zebrafish (Amatruda and Patton, 2008). For example, identification of mutations in ribosomal genes that promote cancers has provided novel insights into how heterozygous loss of ribososomal components promote tumorigenesis (Amsterdam et al., 2004; Lai et al., 2009; MacInnes et al., 2008), and how mutations in genes controlling genome integrity such as Emi1, Separase and B-Myb, can enhance the rate of cancer formation in carcinogen treated or cancer prone animals (Rhodes et al., 2009; Shepard et al., 2005, 2007). More recently, dominant mutations that promote T-cell malignancies and germ-cell tumors in adult fish have been identified (Frazer et al., 2009; Neumann et al., 2009). Zebrafish cancer imaging, stem cell, and cancer treatment strategies are also being pioneered (Goessling et al., 2007; Neumann et al., 2009; Smith et al., 2010; Spitsbergen, 2007). For example, germ-cell tumors in the adult male zebrafish, analogous to human seminomas, are effectively treated by whole body irradiation (Neumann et al., 2009).

**Figure 5:**
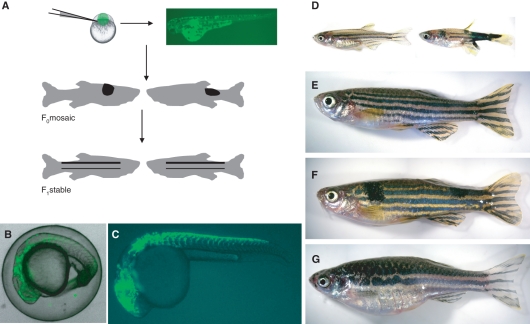
Transgenic melanoma models in zebrafish. (A) Injection of the oncogenic BRAF^V600E^ transgene into the animal pole of the single cell embryo generates mosaic founder (F0) fish that express the transgene in some of the melanocytes, generating ectopic fish-nevi (black spots). Here, the *BRAF* oncogene is expressed under the melanocyte specific *mitfa* promoter: the mosaic expression pattern of melanocytes expressing a *mitfa-GFP* transgene are clearly visible (bright green dots) in the 3-day-old embryo. Some of the mosaic fish will have the transgene in their germ-line, and breeding of these fish generates stable transgenic lines (F_1_) that express BRAF^V600E^ in all neural crest-derived melanocytes. (B) Clear expression of developing neural crest and melanocytes in living embryos (approximately 20 h post-fertilization) expressing the *sox10-GFP* transgene, and (C) the *mitfa-GFP* transgene. (D) A wild type (left) and transgenic HRAS^V12^ (right) 10-week-old zebrafish (about 1 cm in length). The mosaic HRAS^V12^ zebrafish expresses oncogenic *RAS* from the *mitfa* promoter, and shows both ectopic nevi behind the eye and melanoma development in the tail region. (E) An adult wild type zebrafish (3–4 cm in length), (F), a F0 mosaic and (G), a F_1_ stable zebrafish expressing BRAF^V600E^ from the *mitfa* promoter. Note the ectopic black nevi on the mosaic BRAF^V600E^ fish, compared with the expanded top stripe of the stable BRAF^V600E^ fish. Images courtesy of James Lister, Jennifer Richardson, Amy Mitchell and Corina Anastasaki.

A notable feature of the zebrafish system is the ability to generate transgenic animals, allowing visualization of a gene or pathway in the contexts of both embryonic and cancer development (Figure 5). Many of the genes that are involved in melanocyte development are the same genes that are misregulated in melanoma development (Lin and Fisher, 2007), and enhanced transgenic technologies allow for accurate and conditional tissue specific gene expression in the embryo and adult (Curado et al., 2008; Halpern et al., 2008; Kawakami, 2007; Kwan et al., 2007; Yoshikawa et al., 2008). As seen in Figure 5, expression of tissue specific promoters in the melanocyte lineage can assist in visualizing melanocyte progenitors and differentiation in the living embryo, and have been used to promote cancer gene expression in specific tissues. These tools are important in probing the fundamental link between development and cancer, and are a unique asset of the zebrafish system.

### BRAF melanoma models in zebrafish

Although the *Xiphophorus* system makes clear the important role of genetics in melanoma development, it does not provide experimental avenues for facile genome manipulation such as transgenesis, in contrast to zebrafish and medaka. While the natural occurrence of melanoma in zebrafish is rare, knowledge gained from studies of the genetic and environmental *Xiphophorus* melanoma models, coupled with the carcinogen and transgenic induced cancers in zebrafish, led to the use of zebrafish to directly test the relevance of oncogenic BRAF in melanoma. BRAF is a serine/threonine kinase that transduces signals from the upstream RAS kinases to the downstream MEK kinases, as part of the MAP kinase (RAS-RAF-MEK-ERK) signaling pathway (Gray-Schopfer et al., 2007). The MAPK pathway is one of the most frequently activated pathways in cancer, and in melanoma mutations in RAS or BRAF lead to its activation. Sequencing efforts led by the Cancer Genome Project identified BRAF mutations in the majority of the melanoma cell lines and primary tumor specimens (Davies et al., 2002). Of these mutations, over 80% had a specific V600E activating mutation; intriguingly, kinase-impaired BRAF mutations were also identified, and subsequently shown to be potent activators of the MAPK pathway (Dhomen and Marais, 2007). Further analysis of nevi (benign accumulations of melanocytes, commonly known as moles) revealed that the majority of nevi also had BRAF mutations, suggesting that BRAF mutations alone are not sufficient to promote melanoma (Pollock et al., 2003).

Expression of BRAF^V600E^, but not wild-type BRAF, from the melanocyte specific *mitfa* promoter (Dorsky et al., 2000) caused the development of large ectopic melanocytic lesions, as seen in Figure 5. External fertilization of the zebrafish embryo allows for microinjection of transgene DNA constructs at the single-cell stage that will randomly integrate into the genome (Figure 5A). Transgene integration appears to occur during early embryogenesis, and the resulting F0 (founder) fish are mosaic in expression of the transgenic construct (Figure 5A, D, F). Histopathological examination of the BRAF^V600E^ lesions showed that these were not tumorigenic, but rather more closely resembled blue nevi, a darkly pigmented nevus found in human skin; in accordance with the histopathology, these lesions were called fish (f)- nevi (Patton and Zon, 2005; Patton et al., 2005). Genetic crosses of the mosaic BRAF^V600E^ fish produced an F_1_ generation with stable integration of the BRAF^V600E^ transgene (Figure 5G). These fish do not appear to have embryonic melanocyte patterning defects, but during metamorphosis (about 4 weeks of age) develop a distinct widening of the most dorsal melanocyte stripe. Both the BRAF^V600E^ nevi and stable transgenic lines never go on to develop melanoma. Thus, expression of the activating mutation BRAF^V600E^ can promote altered melanocyte proliferation and patterning, but is not sufficient to promote melanoma development.

Unlike with mouse genetics, current technologies in zebrafish do not provide an easy way to engineer site-specific changes by homologous recombination. However, using TILLING (targeting lesions in genomes), and zinc-finger nucleases, mutations in genes of interest can be generated and identified (Amsterdam and Hopkins, 2006; Ekker, 2008). *p53* is the most frequently mutated gene in human cancer, and zebrafish *p53* shares strong sequence and functional homology with human *p53* (Berghmans et al., 2005). Most *p53* mutations are in the DNA binding domain, and sequencing of over 2600 ENU mutagenized F_1_ male fish in the exons that encode the DNA binding domains identified mutations that are orthologous to human *p53* cancer mutations. One of the DNA binding domain mutations, p53^M214K^, causes loss of the apoptotic DNA damage response in embryos and causes peripheral nerve sheath tumors in the adults at about 11 months of age (Berghmans et al., 2005). Although *p53* is not a common mutation in melanoma, the p53 pathway is frequently altered in melanoma (Chin et al., 2006). To test the role of BRAF^V600E^ in a fish with loss of the p53 pathway, BRAF^V600E^ was expressed in the p53^M214K^ line. Nevi developed in the injected fish, some of which progressed to melanoma by 4 months of age. The melanomas were highly invasive, showed genome instability, and could be transplanted to irradiated zebrafish (Patton et al., 2005). Thus, this was the first animal model to demonstrate the role of BRAF^V600E^ in nevi, and that at least one additional genetic mutation is required for melanoma formation (Patton and Zon, 2005; Patton et al., 2005). This model is relevant for mammalian genetics: recently expression of BRAF^V600E^ under the endogenous promoter, specifically within melanocytes, has been shown to promote ectopic melanocyte patterning in mice, and can cooperate with additional genetic mutations to promote melanoma (Dankort et al., 2009; Dhomen et al., 2009).

### RAS melanoma models in zebrafish

Another important oncogene in melanoma is *NRAS*, and mutations in *NRAS* or *BRAF* are detected in almost all human melanoma (Chin et al., 2006; Gray-Schopfer et al., 2007). *NRAS* and *BRAF* mutations are mutually exclusive, such that they are not both found mutated in the same cancers, suggesting that activation of either *BRAF* or *RAS* is sufficient for pathological activation of the MAPK pathway. Approximately one-third of human primary and metastatic melanomas harbor a *RAS* mutation, and *RAS* mutations are found in over half of congenital nevi, almost exclusively at codon 61 (Papp et al., 1999). In mice, activating *RAS* mutations have long been established as important models for the genetics of melanoma (Chin et al., 2006), and the NRAS^Q61K^ mutation can cooperate with *INK4A* or ß-catenin mutations to promote melanoma (Ackermann et al., 2005; Delmas et al., 2007). In zebrafish, expression of NRAS^Q61K^ in melanocytes (from the *mitf*a promoter) promotes dramatic changes in pigmentation patterning, with heavy pigmentation in the dorsal skin and scales, disrupting the characteristic stripe patterning (Dovey et al., 2009). Low-grade melanomas develop in these fish at 1-yr of age, with a dramatic increase in melanoma incidence and age-of-onset when crossed to the *p53* deficient line (Dovey et al., 2009). Like the BRAF^V600E^*p53* melanomas, the NRAS^Q61K^*p53* melanomas share histopathological features with human melanomas. This pathological similarity extends to the molecular pathways: gene set enrichment analysis (GSEA) of microarrays of RNA expression shows the molecular pathways are shared between human and zebrafish melanoma.

HRAS^V12^ is a frequent oncogenic mutation in cancers, and expression in mouse melanocytes has been key to our understanding of how RAS signaling cooperates with mutations in the INK4A-RB or ARF-p53 pathways to promote melanoma (Chin et al., 2006). In zebrafish, expression of HRAS^V12^ from the *mitfa* promoter (Michailidou et al., 2009), from the *kita* promoter (Anelli et al., 2009), or when expressed throughout the fish at low levels (Santoriello et al., 2009), reveals the potential for HRAS^V12^ to promote both ectopic melanocytes and melanoma. In contrast to the BRAF^V600E^ and NRAS^Q61K^ models, HRAS^V12^ fish show ectopic melanocyte patterns during early embryogenesis that can rapidly become melanoma within a few weeks of development (Anelli et al., 2009; Michailidou et al., 2009). As well as sharing similar histopathology with human melanoma, the HRAS^V12^ melanoma models also appear to share epigenomic changes to their genome: global mRNA expression is reduced with the exception of cell cycle genes, and there are visible changes in histone methylation (Anelli et al., 2009). RAS has multiple effector pathways, including the PTEN-AKT pathway, and the combined activation of the MAPK and AKT signaling pathways is one explanation for the potent oncogenic potential of HRAS^V12^. Indeed, dominant-interfering forms of AKT (PI3K) can prevent the activity of HRAS^V12^ in the PTEN-AKT effector pathway, preventing progression of ectopic melanocytes to melanoma (Michailidou et al., 2009). Activation of the MAPK pathway coupled with activation of the AKT signaling pathway is directly relevant to human melanoma: loss of *PTEN* is commonly associated with activating *BRAF* mutations in human melanoma, and genetics in mouse and zebrafish reveal that *PTEN* mutations are required for BRAF^V600E^ mutations to progress from nevi to melanoma (Dankort et al., 2009; J. Richardson, J. den Hertog, E.E.P. unpublished data). Activation of the AKT signaling pathway has also been shown to collaborate with the hedgehog pathway to promote uveal melanoma in zebrafish (Ju et al., 2009).

In cancer, *RAS* and *BRAF* mutations arise de novo in somatic tissues, but germ-line *RAS*, *RAF* and *MEK* mutations have also recently been identified as causing a series of syndromes that share overlapping clinical features. These Cardio-facio-cutaneous (CFC) related syndromes are characterized by specific facial characteristics, heart abnormalities and skin conditions, including enhanced numbers of nevi (Tidyman and Rauen, 2009). The CFC-BRAF allele spectrum includes both kinase-active and kinase-impaired mutant alleles, all of which appear to act as gain-of-function mutations in vivo, and are sensitive to small molecule inhibitors (Anastasaki et al., 2009; Dhomen and Marais, 2007). Both kinase active and kinase impaired BRAF-CFC alleles promote early cell movement phenotypes in zebrafish embryonic gastrulation, and can promote nevi formation in the adult zebrafish (Anastasaki et al., 2009; C. Anastasaki, K. Rauen, E.E.P, unpublished data). Mutations in *HRAS* underlie Costello syndrome, a developmental syndrome characterized by short stature, cancer susceptibility, and heart and mental deficiencies (Tidyman and Rauen, 2009). In zebrafish, ubiquitous expression of low levels of HRAS^V12^ produce adult fish that share characteristics with Costello syndrome, develop melanoma and other cancers, and express the hallmarks of senescence in the heart and brain (Santoriello et al., 2009). One possibility is that tissue specific thresholds to activated RAS, BRAF or MEK expression may underlie differing cellular outcomes, including cell proliferation in melanocytes, movement in early development, and senescence in heart and brain development.

The particular mutation, copy number and tissue specific expression each contribute to the etiology of developmental disease and cancer, including melanoma (Chin et al., 2006; Crowson et al., 2007; Miller and Mihm, 2006). A technical aspect of the transgenic work that is under considerable attention from the zebrafish community is that integrated transgenes are often at variable copy number, and this can influence the physical, cellular and molecular phenotype (Dovey et al., 2009). While the pathology of zebrafish melanoma is relevant to our understanding of human melanoma, new technologies that allow for expression of engineered mutations from the endogenous promoter, as attainable in mice (Dankort et al., 2009; Dhomen et al., 2009), is important to align zebrafish melanoma models, and models of other diseases, with the analogous human condition.

### A new *Xmrk*-medaka melanoma model

As discussed in previous sections, elucidation of the role of *Xmrk* as a potent oncogene in *Xiphophorus* melanoma first established an avenue to investigate the genetic basis of this disease in an experimental animal model. However, *Xiphophorus* is a live-bearing fish, and not amenable to the manipulation of embryos required for such approaches as transgenesis. Circumventing this problem, Schartl and colleagues exploited the ease of using the medaka fish (*Oryzias latipes*) as a genetic model (Schartl et al., 2010). Like zebrafish, medaka is amenable to transgenesis, TILLING, and other sophisticated genetic approaches, and can be accommodated in the laboratory setting. The embryos develop ex utero, are transparent, and many of the promoters are interchangeable between zebrafish and medaka. The genome is sequenced, and like zebrafish, many of the cancer genes and pathways are highly similar to other vertebrates, including humans. Expression of *Xmrk* under the *mitf* promoter resulted in potent melanoma and pigment cell development in melanocytes, as well as other pigment cell types (Schartl et al., 2010). The *Xmrk*-medaka melanomas are highly invasive into the internal organs and spinal cord, and appear metastatic. As often occurs in human melanoma, the more aggressive melanomas contained melanocytes that are often less pigmented and differentiated, compared with melanocytes in the wild type medaka. Melanoma progression was strongly dependent on *Xmrk* dosage: the hemizygous fish developed extended dark black spots at 6–10 weeks, analogous to nevi, and almost half of these pigmented lesions went on to develop into melanoma by 3 months. In contrast, medaka homozygous for the *Xmrk* transgene developed pigmentation changes by 8–10 days post-fertilization that became cancerous within 2–6 weeks with almost complete penetrance (Schartl et al., 2010). Downstream signaling of *Xmrk* has been well studied, and a good understanding of the role of MAPK signaling, PI3 kinase, PLC-gamma, STAT5, FYN and FAK signaling has begun to emerge, as previously discussed. The *Xmrk*-medaka melanoma show activation of the AKT signaling pathway, as well as strong activation of the STAT5 signaling pathway and enhanced MITF protein. As with the HRAS^V12^ zebrafish melanoma models, the potential for *Xmrk* to induce melanoma without cooperating mutations may reflect the upstream signaling potential of RAS to affect a wider series of downstream targets (Schartl et al., 2010). After years of genetics and cell biology, this work definitely demonstrates that *Xmrk* is a highly potent oncogene in vivo.

## Swimming forward: fish as unique tools for melanoma research

With the impressive range of genetic and transgenic models of melanoma progression in three different species of fish, where do we go from here? The unique genetic crosses available in the *Xiphophorus* system provide an opportunity to explore how complex genetic traits, pigmentation and exposure to UV light contribute to melanoma progression, and how pigmentation patterning and oncogenic mutations can be under complex genetic or epigenetic control and sexual selection in the wild. The zebrafish and medaka systems provide the foundation for genetic screening for germ-line variants that influence melanoma pathology, as well as for functional genomic approaches that explore the wealth of the human melanoma genomic data (Greenman et al., 2007; Pleasance et al., 2010). Transparent zebrafish and medaka – used in genetic, transgenic and transplantation approaches – provide unparalleled model systems to observe melanocyte and melanoma biology in vivo. Finally, small molecule screening in zebrafish is driving forward a unique and clinically relevant whole-animal screening approach that is identifying novel and known molecules that affect melanocyte and melanoma biology. Here, we describe some of innovative approaches that are being used to gain new insight into melanoma biology using these distinctive fish models.

### UV light, photocarcinogenesis and melanoma

Sunlight exposure is a critical risk factor for melanoma, and unresolved questions include how gene-environment interactions contribute to nevi and melanoma development, and the UV wavelength dependence of melanoma induction. A recent review (von Thaler et al., 2010) on the relative roles of different wavelengths of UV light in the solar spectrum in inciting melanoma highlights the controversy over whether UVA contributes directly to melanoma causation. Experimental results collected using two *Xiphophorus* hybrid melanoma models have been central to this debate, the Sp-*helleri* hybrid cross (Figure 4B) and the Sp-*couchianus* cross (Figure 4E). Setlow originally used the Sp-*helleri* hybrid melanoma model to demonstrate that UVB irradiation of fry induced melanoma to a significant degree above background incidence (Setlow et al., 1989). This result was confirmed by Nairn et al. (1996b), who also showed that UVB melanoma susceptibility was linked to the *Diff* locus. Setlow and colleagues also used the Sp-*couchianus* model to investigate the wavelength dependence of melanoma induction and reported results indicating that UVA wavelengths were as effective as UVB wavelengths in inducing melanoma (Setlow et al., 1993). UVA is quantitatively more prevalent than UVB (∼ 10-fold) in sunlight incident to the earth’s surface, and Setlow proposed that UVA was therefore more responsible than UVB for inciting melanoma in the human population (Setlow and Woodhead, 1994; Setlow et al., 1993). This assertion was, and remains, highly controversial, since this result has not been replicated in other animal melanoma models studied, as discussed in several recent reviews (Bennett, 2008; Lund and Timmins, 2007; Noonan et al., 2003). In support of the UVA induction hypothesis a study was recently published using the same Sp-*couchianus* model to investigate photosensitization of melanin as a possible mechanism for melanoma formation (Wood et al., 2006). Electron paramagnetic resonance was used to monitor UV induction of reactive melanin radicals in pigmented skin. The action spectrum for melanin-sensitized generation of reactive radicals approximately tracked the action spectrum for melanoma formation reported by Setlow et al. (1993), consistent with a role for UVA in melanoma causation through a free radical mechanism, requiring the presence of melanin. However, a very recent study (D.L.M, A. Fernandez, R.S.N., R. Garcia, L. Panniker, D. Trono, H. Thames, I. Gimenez-Conti, submitted) attempted to reproduce Setlow’s melanoma induction results using larger numbers of Sp-*couchianus* BC_1_ hybrids and concluded that UVA did not induce melanomas above the background incidence in this model, but that UVB was effective. Also, UVA fails to induce melanoma in BC_1_ hybrids from the original UVB-inducible Sp-*helleri* model (R.S.N, unpublished results). These results are consistent with mouse studies in which UVB, but not UVA, induced melanoma (De Fabo et al., 2004). However, this controversy is likely to continue, especially since suntanning beds have widespread use in Europe and North America, and advertise their ‘safety’ on the basis of emitting predominantly UVA light. Investigation in the future of UV inducibility of melanoma in other *Xiphophorus* crosses, and the transgenic models recently developed in zebrafish and medaka, can play a significant role in resolving this issue.

In fact, the wealth of experience in UV-induced melanoma protocols developed by the *Xiphophorus* community is now being translated to the zebrafish system (Zeng et al., 2009). As in mammalian cells, the UV DNA damage response involves p53, and the p53^M214K^ genetic line fails to initiate an apoptotic-DNA damage response after UV treatment (Zeng et al., 2009) Interestingly, it appears that the UV damage response may be developmentally regulated in zebrafish embryos (Dong et al., 2007; Zeng et al., 2009). However, less work has been done on the effects of UV light on adult zebrafish, and studies that are analogous to human environmental UV exposure conditions will be important. Recently, exposure of adult zebrafish skin to UV light has been shown to activate a phospho-H2AX DNA damage response, and *p53* deficient zebrafish have a decreased ability to promote repair of UV induced DNA damage in their skin (Zeng et al., 2009). Early UV treatment results of genetic and transgenic cancer prone lines suggest that some zebrafish genetic backgrounds may be sensitive to melanocyte changes after UV treatment (Z. Zeng, D.L.M., E.E.P., unpublished data).

### Gene modifiers of melanoma progression

Many heritable traits are polygenic, and while studies of *Xiphophorus* melanoma have revealed strong genetic determinants of melanoma susceptibility, other genes having more modest effects are likely also to be involved in modulating the melanoma susceptibility phenotype. The existence of several *Xiphophorus* backcross hybrid melanoma models exhibiting different melanoma susceptibilities, as discussed (see Figure 4), offers a unique opportunity to apply quantitative trait linkage (QTL) analysis to identify additional genes that modify melanoma formation and progression. The underlying basis of using genetic markers to detect QTL is genetic linkage; there tends to be less meiotic recombination between regions of a chromosome that lie close to one another than for those lying far apart. Thus, alleles at a polymorphic marker locus and a polymorphic QTL that lies close to it will tend to segregate together at meiosis. The closer together a QTL and flanking marker are, the tighter this intergenerational association will be. Since *Xiphophorus* melanoma models are generated through interspecies hybridization, F_1_ hybrids have highly elevated heterozygosity throughout the genome, and backcross progeny exhibit a wide range of multilocus genotypes not found in either parental species. In primary segregating populations generated from genetic backcrossing (i.e., BC_1_ hybrids) the association of specific marker-locus alleles and QTL alleles derives directly from the haplotypes of the parental species. Thus the underlying basis for mapping QTLs for complex traits in this situation is to detect a correlation between marker allele and phenotypic state of the complex trait. This is conceptually similar to seeking direct associations between allelic variants and phenotypic states in genome-wide association studies. Specifically, In BC_1_ hybrids, representing only a single generation of meiotic recombination, residual linkage disequilibrium between a QTL and a marker locus will reflect the genetic distance between them. Interspecific hybridization and backcrossing thus provide a powerful strategy to construct genetic linkage maps with dense coverage and to identify chromosomal regions that harbor QTLs that influence physical and physiological phenotypes – including melanoma susceptibility. The recent availability of sophisticated genetic resources for *Xiphophorus,* such as BAC libraries (e.g., Froschauer et al., 2002; Walter et al., 2006), further enhances the strength of this approach. The *Xiphophorus* hybrid melanoma models are therefore ideally suited to the application of QTL analysis for revealing genes that may individually exert modest effects on melanoma susceptibility and/or progression. This general approach can also be extended to other genes that may be important in melanoma, such as DNA repair genes, in *Xiphophorus* models (Mitchell et al., 2007).

In the zebrafish melanoma models, testing the function of new and known genes that collaborate with BRAF to promote melanoma progression and invasion is an important next step. Len Zon and colleagues are screening melanoma relevant genes for enhanced melanoma progression in the BRAF^V600E^*p53* model, which will provide insight into novel melanoma progression pathways (L. Zon, personal communication). Direct testing of additional genetic lines of the PTEN pathway and the MITF pathway is also underway, revealing new understanding of how cooperating mutations collaborate with BRAF^V600E^ in melanoma development and pathology (J. Richardson, J. den Hertog, J. Lister, E.E.P., unpublished data). One the most important aspects of the *Xmrk*-medaka model is the identification of genetic modifiers of pigment cell tumor incidence, pathology and tumor spectrum (Schartl et al., 2010). While laboratory fish are not clones, very often lines of fish are maintained that are derived from a small founder population. In the HB32C background, *Xmrk* expression primarily causes highly invasive melanomas. In contrast, in the *Carbio* background, a non-inbred line and of mixed genetic background, *Xmrk* expressing fish rarely develop melanoma, and instead develop almost exclusively exophytic xanthoerythrophoromas (tumors in the yellow and red pigment cells), that break into the underlying musculature only at the terminal stages. With the loss of *p53* in the *Carbio* background, the tumor spectrum in the *Xmrk*-medaka fish changes, with development of fast growing nodular melanomas. Finally, in the *albino* (*i-3*) background, weakly pigmented melanomas develop in about a third of the fish, while almost half of the fish develop uveal melanomas. These studies demonstrate the ability of genetic context and background to shape the tumor spectrum, size and pathology, and provides a framework for future genetic screens and crosses to identify genetic modifiers of melanoma pathology.

### Melanoma and the microenvironment

While intensive efforts are concentrated on understanding the genetic and epigenetic conditions that cause a melanocyte to transform to melanoma, recent work by the Hendrix laboratory and others has developed the zebrafish as a ‘biosensor’ to explore the bidirectional signaling of melanoma cells within the environment of the whole embryo (Hendrix et al., 2007; Topczewska et al., 2006). Aggressive melanoma cells are highly motile and adopt characteristics of de-differentiated, multipotent neural crest progenitors that can respond to and influence cells in their environment (Hendrix et al., 2007). This plasticity is also a characteristic of embryonic stem cells: in the developing embryo neural crest cells give rise to multiple cell types that actively migrate and invade embryonic tissues to arrive at the skin and fully differentiate into melanocytes (White and Zon, 2008). The microenvironment plays an important role in promoting the behavior and fate of both embryonic stem cells and melanoma cancer cells, and understanding the cellular communication between cells and their environment is at the intersection of both developmental and cancer biology (Kasemeier-Kulesa et al., 2008).

As zebrafish embryos are transparent, and develop externally, fluorescently labeled human cancer cell lines can be injected into the embryo and assessed for influence of the environment on the melanoma cells, and *vice versa* (Hendrix et al., 2007). By injection of melanoma cancer cell lines with varying degrees of metastatic potential into the zebrafish embryo, Mary Hendrix and colleagues identified an aggressive melanoma cancer line that could influence the development of the surrounding embryonic cells (Topczewska et al., 2006). Injection of melanoma cells into the animal pole of the early developing embryo (blastula-stage, at 3hpf) induced the zebrafish embryo to develop an ectopic cranial outgrowth. Similarly, injection of the cells into the margin of the blastula induces the formation of a secondary axis. Interestingly, some of the melanoma cells injected into later stage embryos also appear to be able to be reprogrammed by their environment; while human melanoma cells can survive in the adult zebrafish, their cancerous phenotypes are suppressed (Lee et al., 2005). The axis reorganizing activity of the metastatic melanoma cells was identified as the morphogen, Nodal. In culture, reduction of Nodal activity restores the differentiated melanocyte phenotype (e.g*.,* expression of tyrosinase), while eliminating the trans-differentiated phenotype. Thus, Nodal is a novel melanoma-dependent pathway that both shapes the embryonic surrounding and maintains melanoma plasticity. Importantly, inhibition of Nodal causes a reduction of tumorigenicity in the mouse, and has been accurately identified as a prognostic biomarker for melanoma (Strizzi et al., 2009). Nodal had not previously been implicated in melanoma progression, and the use of zebrafish as a ‘biosensor’ has successfully identified Nodal as a key signaling pathway for melanoma, and a potential therapeutic target.

Zebrafish and medaka are also being used as a xenograft models to study cancer cell proliferation, migration, and angiogenesis (Hasegawa et al., 2009; Nicoli and Presta, 2007; Stoletov and Klemke, 2008). As xenograft models, fish have the advantages of unprecedented imaging quality and are highly amenable to cost-effective pharmacological testing (Hasegawa et al., 2009; Stoletov and Klemke, 2008; Stoletov et al., 2007). Human melanoma cells injected into 2-day-old zebrafish embryos can survive, proliferate, migrate, form tumor-like masses and induce a robust angiogenic response (Haldi et al., 2006; Nicoli et al., 2007, 2008). Human cancer cells can also be injected later in development (e.g., 30 days), and while the immune system must be chemically suppressed, the organs and vascular system are already developed, uncoupling the effects of development on the xenograft (Stoletov and Klemke, 2008; Stoletov et al., 2007). In medaka, inbred lines allow for transplantation of syngeneic melanoma cancer cell lines into the adult host without irradiation, allowing for the following of in vivo imaging of cancer cells at all stages of development (Hasegawa et al., 2009). The dynamic interactions between cancer cells and host tissues have been captured using confocal microscopy on transgenic zebrafish expressing GFP in the vasculature, enabling exceptional intravital imaging of labeled cancer cells invading and remodeling the host vasculature (Stoletov et al., 2007). Importantly, gene knockdown, genetic engineering technologies, and direct injection of proteins and chemicals can modify both the zebrafish host and/or the cancer cells (Nicoli and Presta, 2007; Stoletov and Klemke, 2008). For example, B16 human melanoma cells injected into a 2-day-old zebrafish embryo can promote a robust angiogenic response in the zebrafish that can be reduced by exposure to chemical inhibitors of FGF and VEGF receptors. Similarly, knockdown of the cell-cell adhesion molecule, VE-cadherin, in the zebrafish embryo can prevent tumor-induced angiogenesis without altering normal vessel development (Nicoli et al., 2007).

**Figure 6:**
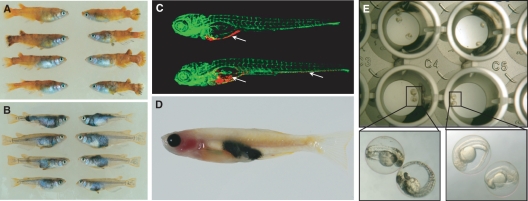
Creative approaches to studying melanocytes and melanoma in fish. Genetic modifiers can alter pigment cell tumor spectrum in the *Xmrk* medaka model: (A) *Xmrk* in the *Carbio* line promotes exophytic yellow and red cell tumors, but (B) with the loss of *p53* in the *Carbio* line there is a dramatic shift in the tumor spectrum, and the fish succumb to endophytic, highly invasive melanoma. In (C) deRed labeled human melanoma cells are clearly visible at the yolk sac (arrow, top fish) of an 8-day-old zebrafish embryo. The vasculature is highly visible through expression of the *fli1-GFP* transgene. An invasive melanoma cell line begins to invade the developing intestinal bulb and circulates in the blood vasculature (arrows; bottom fish). (D) The *Casper* zebrafish lacks body pigment: darkly pigmented transplanted melanoma cells can be clearly seen in the internal body of the zebrafish. (E) Two-day-old zebrafish embryos, still in their chorion (permeable shell) are arrayed in the wells of a 96-well plate. Each well contains a small molecule dissolved in 300 μl of fish-water: the embryos in well C3 (left) are not affected by the compound in the well, and have the normal melanocyte pigmentation pattern, while the compound in well C4 (right) prevents normal melanocyte pigmentation and the embryos are white. Images by Manfred Schartl, Shuning He, Ewa Snaar-Jagalska, Richard White, Len Zon, and E.E.P.

While zebrafish and medaka early embryos are transparent, the pigment of juvenile and adult fish obscure internally labeled cells. New transparent medaka and zebrafish provide a unique window into the development of tissues during development and during adulthood (Figure 6). For example, in medaka, GFP reporter expression in the germline allows for visualization of the developing testis and ovary from embryogenesis to adulthood, and the detailed and continued maturation of the ovary after spawning in the adult (Wakamatsu et al., 2001). In zebrafish, Zon and colleagues have also recently generated a transparent adult zebrafish named *Casper* that lacks body pigment cells through mutation of *mitf*, and an as of yet unidentified mutation *roy* (White et al., 2008). This transparent fish allows for the impressive visualization of labeled transplanted cells, such as GFP-labeled marrow cells after irradiation ablation of the hematopoietic cells, or the growth and metastasis of pigmented melanoma cells (Figure 6). The naturally transparent embryo and the adult fish provide unique resources for the study of both engrafted and endogenous melanoma cell characteristics, stem cells and microenvironment interactions in a living animal.

### Small molecule screening in fish

The small size of the developing zebrafish and medaka makes them ideal organisms to study the effects of small molecules on melanocyte development and melanoma models. The fundamental link between development and cancer means that small molecules that alter melanocyte biology and regeneration may be relevant to our understanding of melanoma development (White and Zon, 2008). We and others have performed small molecule screens, and have identified chemical compounds that interfere with specific aspects of melanocyte biology, including melanocyte development, migration, pigmentation, and survival (O’Reilly-Pol and Johnson, 2009; White and Zon, 2008; H. Ishizaki, R. Kelsh, E.E.P., unpublished data). In the developing zebrafish, melanocytes become visible by approximately 28 h post-fertilization (Kelsh et al., 2009), and fluorescent reporter lines allow for neural crest progenitors and unpigmented melanocytes to be visualized in the living embryo (Figure 5). Multiple embryos can easily be arrayed into each well of a 96-well plate in about 300 μl of embryo water containing specific chemicals, as shown in Figure 6(E) (Kaufman et al., 2009). The high fecundity of the zebrafish allows for hundreds to thousands of chemicals to be screened each week in an academic laboratory.

Stephen Johnson and colleagues have used small molecules to temporally control melanocyte cell death, and through chemical and genetic screens have identified compounds that control the recruitment and development of a melanocyte stem cell population (O’Reilly-Pol and Johnson, 2009; White and Zon, 2008). Chemical control of these cell types and of the pathways that modulate their development complement the genetic mutants that alter melanocyte biology, and provide novel hypotheses to test which cell types and pathways contribute to melanocyte and melanoma development. The identified chemicals may also be valuable drug-like leads: screening of clinically approved drugs on zebrafish embryos has identified prostagalandin as an important regulator of hematopoietic stem cells in embryonic and adult fish and in mice, and is currently in clinical trial for enhancing hematopoietic stem engraftment after marrow depletion (North et al., 2007; L. Zon, personal communication). Treating fish with small molecules is not limited to the embryonic stages: adult fish can be directly immersed in chemical treatment water, or through chemical injection into the retro-orbital of the eye (Pugach et al., 2009). Small molecules are also being screened on embryos that have been transplanted with fluorescent human cancer cells to modulate proliferation, migration and angiogenesis, as discussed above (Figure 6C). These studies hold promise for identifying new and targetable pathways in melanocyte development and melanoma, and also microenvironment pathways that might directly alter melanoma progression.
